# Dosimetric Study of Biaxially Rotational Dynamic Radiation Therapy for Hippocampal-Sparing Whole Brain Irradiation

**DOI:** 10.3390/cancers17121949

**Published:** 2025-06-11

**Authors:** Kouta Hirotaki, Kenji Makita, Masaki Nakamura, Masashi Wakabayashi, Satoe Kitou, Takashi Ninomiya, Masashi Ito

**Affiliations:** 1 Department of Radiological Technology, National Cancer Center Hospital East, Chiba 277-8577, Japan; khirotak@east.ncc.go.jp (K.H.); sfukuhar@east.ncc.go.jp (S.K.); masasito@east.ncc.go.jp (M.I.); 2 Department of Radiation Oncology, National Cancer Center Hospital East, Chiba 277-8577, Japan; masanaka@east.ncc.go.jp; 3 Department of Radiation Oncology, NHO Shikoku Cancer Center, Ehime 791-0280, Japan; 4 Biostatistics Division, Center for Research Administration and Support, National Cancer Center Hospital East, Chiba 277-8577, Japan; mawakaba@east.ncc.go.jp; 5 Department of Thoracic Oncology and Medicine, NHO Shikoku Cancer Center, Ehime 791-0280, Japan; ninomiya.takashi.se@mail.hosp.go.jp

**Keywords:** biaxially rotational dynamic radiation therapy, volumetric modulated arc therapy, OXRAY, whole brain irradiation, hippocampal sparing

## Abstract

**Objectives:** Although hippocampal-sparing whole-brain irradiation (HS-WBI) offers potential neurocognitive benefits, it poses challenges in treatment planning. This study aimed to compare the dose distributions of biaxially rotational dynamic radiation therapy (BROAD-RT) with a novel O-ring-type linear accelerator (OXRAY) and conventional non-coplanar volumetric modulated arc therapy (VMAT) planning (Conv-VMAT) in HS-WBI treatment plans. **Methods:** This study included 10 patients with brain metastases from lung cancer at our institution. The hippocampus was contoured using gadolinium-based contrast-enhanced magnetic resonance imaging, and hippocampal-sparing regions were created using a 5 mm margin around the hippocampus. Two virtual plans (BROAD-RT and Conv-VMAT) with 30 Gy in 10 fractions were created to compare the dose distributions in the planning target volume (PTV), hippocampus, eyes, and lens. All plans were analyzed using a paired *t*-test. **Results:** The mean (standard deviation [SD]) hippocampus-Dmax, -Dmean, -D100%, and -V10 were 11.10 (0.61), 7.95 (0.20), 7.01 (0.19), and 0.42 (0.34) for BROAD-RT and 16.10 (0.57), 9.89 (0.75), 8.24 (0.34), and 39.05 (25.89) for Conv-VMAT, respectively. All hippocampal parameters were significantly better with BROAD-RT than with Conv-VMAT (*p* < 0.01). The PTV-D98, -D50, -D2, -V35, and -homogeneity index did not exhibit significant differences between BROAD-RT and Conv-VMAT. Although lens-Dmax was significantly better in BROAD-RT than in Conv-VMAT (*p* < 0.01), no significant differences were observed in the eye-Dmax and chiasm-Dmax between BROAD-RT and Conv-VMAT. The mean (SD) BROAD-RT beam delivery time was 313.60 (34.91) s. **Conclusions:** BROAD-RT improved hippocampal sparing with acceptable PTV coverage and PTV homogeneity in HS-WBI planning. In addition, BROAD-RT has a clinically acceptable treatment duration.

## Simple Summary

In patients with brain metastasis and adequately long-term survival, whole brain irradiation (WBI) leads to progressive memory loss and dementia. In this study, hippocampus-sparing WBI (HS-WBI), which has potential benefits in neurocognitive preservation, was planned using a novel radiation treatment device, biaxially rotational dynamic radiation therapy (BROAD-RT) using a novel O-ring-type linear accelerator. BROAD-RT improved hippocampal sparing with acceptable planning target volume coverage with a clinically acceptable treatment time. These data suggest that BROAD-RT is a treatment device that is clinically feasible in terms of both dose distribution and treatment time for HS-WBI and may be a possible therapeutic option.

## 1. Introduction

Brain metastases are the most common brain tumors in adults, accounting for more than half of all brain tumors. In patients with metastatic tumors, brain metastases occur in 10–30% of adults and 6–10% of children [1,2,3,4]. Patients with brain metastasis have extremely poor survival (median survival time, 1 month) if they do not receive any treatment for brain metastasis [5,6]. However, recently, the Graded Prognostic Assessment was developed as a prognostic scoring system for patients with brain metastases treated with an optimal treatment strategy [7,8]. Based on their scoring system, patients with a good prognostic score have a predicted overall survival of approximately over 1 year [7,8].

Various treatments, such as medication, surgery, and radiotherapy, including whole brain irradiation (WBI) and stereotactic radiotherapy, for brain metastases are known to improve neurological symptoms, decrease tumor growth, and extend prognosis [9]. WBI is the most commonly used method for patients with multiple brain metastases because of its usefulness in controlling neurological symptoms and reducing disease burden [10,11,12]. However, various late adverse events of WBI, which are considered secondary to vascular injury and demyelination, sometimes occur and can be irreversible and progressive when patients treated with WBI have an adequately long survival time [13]. Of the late adverse events of WBI, progressive memory loss and dementia are some of the most avoidable events [14,15]. Therefore, specific WBI with neurocognitive preservation is desirable for patients with a good prognosis.

RTOG 0933, which evaluated the effect of the hippocampus-sparing WBI (HS-WBI) on neurocognitive function, demonstrated its potential benefits in neurocognitive preservation [16]. These findings were subsequently validated by the NRG CC001 study, and several studies have suggested the usefulness of HS-WBI in evaluating neurocognitive function [17,18,19]. Therefore, recently, the guidelines of the American Society of Clinical Oncology, Society for Neuro-Oncology, and American Society for Radiation Oncology on the treatment for brain metastases recommended that the HS-WBI should be used in patients who are treated with WBI, have no hippocampal metastatic lesions, and with an expected survival time of ≥4 months [9].

Based on these studies, many dosimetric approaches have been assessed for various treatment modalities to improve the dose distribution of HS-WBI [20,21,22,23,24]. Among these, Rong et al. suggested that helical tomotherapy was a favorable modality for HS-WBI because of its superior homogeneity index (HI) compared with that of intensity-modulated radiation therapy (IMRT) or volumetric modulated arc therapy (VMAT); however, helical tomotherapy has a long treatment duration. Recently, OXRAY (Hitachi, Ltd., Tokyo, Japan), an O-ring-type novel linear accelerator system, was developed as the successor to Vero4DRT (Hitachi, Ltd., Tokyo, Japan) [25,26,27,28,29,30,31]. The O-ring synchronizes the gantry rotation, enabling non-coplanar VMAT without moving the patient’s couch. In HS-WBI planning, this novel OXRAY system has the potential to improve various dose–volume parameters without worsening the treatment duration. Therefore, in this study, we evaluated the impact of biaxially rotational dynamic radiation therapy (BROAD-RT) using the OXRAY system to improve the dose distribution for HS-WBI without worsening treatment duration.

## 2. Materials and Methods

### 2.1. Patients

Ten consecutive patients with brain metastases who underwent radiotherapy at our institution between July 2023 and July 2024 were assessed. The present study was approved (No. 2018-076) by the Ethics Committee of our institution (National Cancer Center Hospital East, Kashiwa, Japan), and opt-out consent was obtained owing to the retrospective nature of the study.

The head of each patient was immobilized using a thermoplastic shell. Simulation computed tomography (CT) datasets were acquired using Aquilion One (Canon Medical Systems, Tochigi, Japan), with an image slice thickness of 2 mm.

### 2.2. Contouring of the Target and Organs at Risk

All structures were contoured by radiation oncologists and reviewed by other oncologists. The hippocampus was identified using contrast-enhanced T1-weighted magnetic resonance imaging. The hippocampal structure was contoured using the hippocampal atlas for radiotherapy [32]. The hippocampal-sparing region for dose fall-off was contoured with the hippocampus plus a 5 mm margin. The planning target volume (PTV) was defined as the entire brain, excluding the hippocampus and hippocampal-sparing region. Other evaluated organs at risk, such as the eyes, lens, optic nerves, and optic chiasm, were contoured based on simulation CT. A summary of these structures is presented in Table 1.

### 2.3. OXRAY System

**Figure 1:**
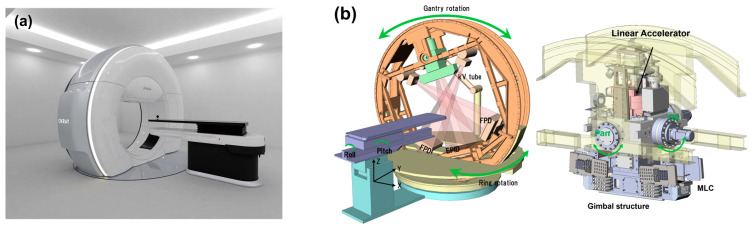
Overview of the OXRAY system. (**a**) Photograph showing the external appearance of the novel linear accelerator, OXRAY. The system features an O-ring gantry structure capable of synchronized biaxial rotation, which enables dynamic noncoplanar beam delivery without couch rotation. (**b**) Schematic diagram illustrating the main components of the OXRAY system, including the robotic couch with five-axis movement, dual-CBCT imaging units, ring gantry, and gimbal mechanism for pitch adjustment. These integrated features enable precise image-guided radiation therapy and flexible, collision-avoidant beam trajectory control. OXRAY, next-generation O-ring–type linear accelerator; CBCT, cone-beam computed tomography.

A photograph and schematic diagram of the novel OXRAY linear accelerator are shown in Figure 1. OXRAY is a next-generation O-ring–type linear accelerator that features a robotic couch with five-axis movement and a ring gantry structure equipped with two X-ray tubes and a rotating gantry. The two orthogonally positioned X-ray tubes enable simultaneous orthogonal imaging, allowing for dual cone beam CT (dual-CBCT) acquisition with a rapid imaging time of approximately 15 s. This feature enhances image guidance efficiency and accuracy. The internal multileaf collimator is composed of 2.5 mm leaves in the central region and 5 mm leaves in the peripheral region, offering dosimetric precision comparable to that of high-end linear accelerators. The system supports a variable dose–rate delivery. The most distinctive feature of the OXRAY system is its ability to perform dynamic trajectory irradiation through a synchronized rotation of the gantry and O-ring. This enables the creation of complex, noncoplanar beam trajectories without requiring couch rotation. The O-ring rotation is mechanically capable of ±60°, allowing for a flexible noncoplanar beam–angle selection within collision–avoidance constraints. Furthermore, a gimbal mechanism installed on the gantry enables an additional ±3° of pitch adjustment in the craniocaudal direction, further expanding the versatility of achievable trajectories.

### 2.4. Virtual Planning and Evaluation

**Figure 2:**
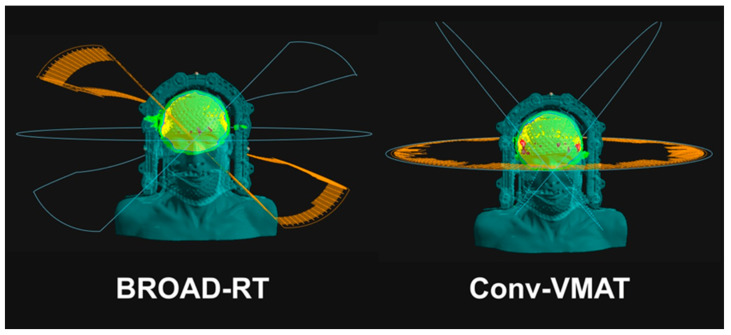
Ring angle synchronized with the gantry angle using BROAD-RT and Conv-VMAT. BROAD-RT, biaxially rotational dynamic radiation therapy; Conv, conventional; VMAT, volumetric modulated arc therapy.

Two virtual radiotherapy plans were delivered at 30 Gy in 10 fractions (D95 = 100%; 95% of the PTV received 100% of the prescribed dose) to the PTV. The two virtual radiotherapy plans were as follows: (1) BROAD-RT with OXRAY (two arcs with continuous modulating ring angles at nine modulation points with a 6 MV photon beam) and (2) conventional non-coplanar VMAT with TrueBeam (Varian Medical Systems, Palo Alto, CA, USA; Conv-VMAT, four arcs and collimator angles of 345° and 15° with a 6 MV photon beam; Figure 2).

All the plans were created by well-trained radiation oncologists and medical physicists. Treatment was planned using the RayStation planning system (Raysearch, Stockholm, Sweden). The Collapsed Cone Version 5.8 algorithm was used for calculations. The dose–volume parameters evaluated were as follows: PTV-D98 (Gy), PTV-D50 (Gy), PTV-D2 (Gy), PTV-V35 (%), PTV-HI, hippocampus-Dmax (Gy), hippocampus-Dmean (Gy), hippocampus-D100% (Gy), hippocampus-V10 (%), eye-Dmax (Gy), lens-Dmax (Gy), chiasm-Dmax (Gy), and parotid grands-Dmean (Gy). Irradiation time and total monitor unit (MU) values were also evaluated. The dose constraints for optimization are shown in Table S1 and information on the devices used for treatment planning is summarized in Table S2.

### 2.5. Statistical Analysis

Descriptive statistics, such as the mean and standard deviation (SD), were used to summarize the dose distributions. The paired *t*-test was used to compare the two radiotherapy plans (BROAD-RT and Conv-VMAT). Statistical significance was set at *p* < 0.05. All statistical analyses were performed using SAS (version 9.4; Cary, NC, USA).

## 3. Results

### 3.1. PTV Dose–Volume Parameter Comparison Between BROAD-RT and Conv-VMAT Planning

The mean (SD) of PTV-D98, -D50, -D2, -V35, and -HI was 27.95 (0.54), 31.65 (0.26), 33.55 (0.49), 0.22 (0.26), and 0.18 (0.02) for BROAD-RT and 28.21 (0.48), 31.58 (0.44), 33.26 (0.66), 0.17 (0.30), and 0.16 (0.03) for Conv-VMAT, respectively. Regarding the target coverage dosimetric parameters, no significant differences were observed between the BROAD-RT and Conv-VMAT groups (Table 2).

**Figure 3:**
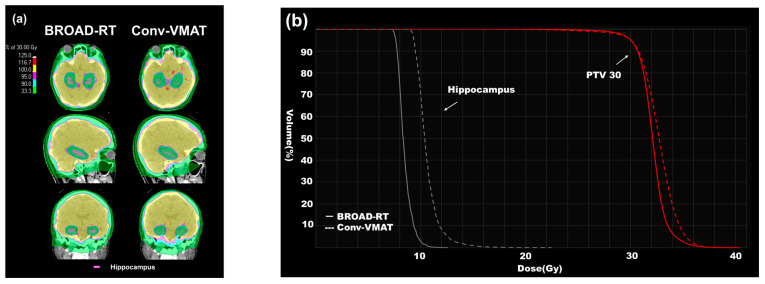
Differences in dose distribution (BROAD-RT vs. Conv-VMAT). (**a**) CT images displaying dose distribution of radiotherapy to HS-WBI (30 Gy in 10 fractions). The red line indicates the hippocampus. BROAD-RT achieved a sharp dose reduction in the hippocampal region compared to Conv-VMAT. (**b**) Dose–volume parameters of the PTV and hippocampus. Red line, PTV; white line, hippocampus. Compared to the Conv-VMAT plan, the BROAD-RT plan improved the important parameters of the hippocampus without deteriorating the PTV coverage. PTV, planning target volume; BROAD-RT, biaxially rotational dynamic radiation therapy; Conv, conventional; VMAT, volumetric modulated arc therapy; CT, computed tomography; HS-WBI, hippocampus-sparing whole brain irradiation.

Examples of BROAD-RT and Conv-VMAT dose distributions are shown in Figure 3a,b.

### 3.2. Hippocampus Dose–Volume Parameter Comparison Between BROAD-RT and Conv-VMAT Planning

The mean (SD) of the hippocampus-Dmax, -Dmean, -Dmin, and -V10 was 11.10 (0.61), 7.95 (0.20), 7.01 (0.19), and 0.42 (0.34) for BROAD-RT and 16.10 (0.57), 9.89 (0.75), 8.24 (0.34), and 39.05 (25.89) for Conv-VMAT, respectively. BROAD-RT influenced the hippocampus-Dmax, -Dmean, -Dmin, and -V10 improvements compared to Conv-VMAT (*p* < 0.01, Table 2 and Figure 3a,b).

### 3.3. Other Dose–Volume Parameter Comparisons Between BROAD-RT and Conv-VMAT Planning

The mean (SD) of the eye-Dmax, lens-Dmax, and chiasm-Dmax was 16.17 (1.68), 3.76 (0.16), and 34.28 (1.04) for BROAD-RT and 15.23 (1.58), 4.26 (0.32), and 34.66 (1.30) for Conv-VMAT, respectively. BROAD-RT influenced lens-Dmax improvement compared to Conv-VMAT (*p* < 0.01, Table 2), but did not influence eye-Dmax and chiasm-Dmax improvement (*p* = 0.20 and 0.50, respectively; Table 2). In addition, the mean (SD) of the parotid glands-Dmean was 4.83 (0.35) for BROAD-RT and 4.35 (0.83) for Conv-VMAT, and the difference was significant (*p* = 0.03, Table 2).

### 3.4. Beam Delivery Time Comparison Between BROAD-RT and Conv-VMAT Planning

The mean (SD) total MUs were 1743.70 (151.44) for BROAD-RT and 1222.71 (280.20) for Conv-VMAT. The total MU was significantly higher in the BROAD-RT plan than in the Conv-VMAT plan (*p* < 0.01, Table 2). The beam delivery time, excluding couch rotation time, was 313.60 s (34.91) for BROAD-RT and 202.50 s (14.46) for Conv-VMAT. A significant difference was observed in the beam delivery time between the BROAD-RT and Conv-VMAT plans (*p* < 0.01; Table 2).

## 4. Discussion

This study investigated the usefulness of BROAD-RT on dose distribution for HS-WBI. Compared with Conv-VMAT, BROAD-RT for HS-WBI planning improved hippocampal dose–volume parameters, such as the hippocampus-Dmax, -Dmean, -Dmin, and -V10, without worsening the PTV dose distribution. In the BROAD-RT plan, the beam delivery time (excluding the couch rotation time) was approximately 100 s longer than that in the Conv-VMAT plan.

The incidence of brain metastases in the hippocampal region (hippocampus plus a 5 mm margin) is estimated to be very low (8.6%) [33]. Therefore, reduction in the hippocampal dose by HS-WBI is safe and has a positive impact on the patients’ quality of life because of the prevention of cognitive dysfunction. In this study, the BROAD-RT plan significantly improved the hippocampal dose without worsening the PTV dose distribution compared with Conv-VMAT. This suggested that HS-WBI with BROAD-RT seemed to be preferable over Conv-VMAT for WBI to prevent cognitive dysfunction. The feasibility of HS-WBI has been investigated using various treatment devices [20,21,22,23,24]. Among these, Rong et al. suggested that helical tomotherapy was a favorable modality for HS-WBI because of its superior HI compared to that of IMRT or VMAT [20]. Similarly, Gondi et al. demonstrated that helical tomotherapy exhibits a sharper dose fall-off in the hippocampal region. Nevertheless, both helical tomotherapy and linear accelerator-based IMRT achieved acceptable target coverage and homogeneity [23]. In this study, although we could not compare with the helical tomotherapy plan because we did not have a helical tomotherapy treatment planning device at our institution, comparisons in the literature showed that the dose–volume parameters of BROAD-RT and helical tomotherapy seemed to be similar or that of BROAD-RT seemed to be slightly better than that of helical tomotherapy (Table S3) [20,23]. Therefore, we consider that BROAD-RT is a useful treatment modality for HS-WBI. Furthermore, recent clinical trials have investigated the utility of hippocampal-sparing techniques for prophylactic cranial irradiation in patients with small-cell lung cancer [34]. The effectiveness of BROAD-RT in HS-WBI may also apply to HS–prophylactic cranial irradiation.

Although helical tomotherapy is a favorable modality for HS-WBI because of its hippocampal-sparing ability without worsening the PTV coverage, it has a very long treatment duration (approximately 18 min) [23]. In this study, the beam delivery time of BROAD-RT was significantly longer than that of Conv-VMAT (approximately 5 vs. 3 min, *p* < 0.01). However, the beam delivery time of BROAD-RT appears to be acceptable and is remarkably shorter than that of helical tomotherapy.

Yokoyama et al. compared Halcyon-based VMAT with helical tomotherapy for HS-WBI and showed that Halcyon-based VMAT can provide irradiation with significantly shorter treatment durations than those with helical tomotherapy [24]. However, the hippocampus-Dmax of Halcyon-based VMAT was worse than that of helical tomotherapy (hippocampus-Dmax of Halcyon-based VMAT vs. helical tomotherapy: 14.32 Gy vs. 12.63 Gy). This also seemed to be similar when the hippocampus-Dmax of Halcyon-based VMAT was compared with that of BROAD-RT (hippocampus-Dmax = 11.10 Gy) in our study. Overall, we consider that BROAD-RT is an effective treatment for HS-WBI. Furthermore, the beam delivery time in our study did not include the couch rotation time, which is required for a noncoplanar treatment plan with the non-BROAD-RT planning device. When the total treatment time—including couch rotation and image-guided radiation therapy procedures such as CBCT acquisition and image registration—is considered, the Conv-VMAT plan often requires an additional 1–2 min in clinical practice. Thus, when these non-beam delivery times are included, the difference in treatment time between BROAD-RT and Conv-VMAT may be small. In addition, a representative BROAD-RT workflow consists of approximately 2 min for patient setup, 15 s for CBCT acquisition, 1–2 min for image registration (depending on operator skill), and 5 min for beam delivery. Thus, the entire procedure can be completed within approximately 15 min. Therefore, we consider that BROAD-RT with OXRAY is a feasible treatment device for HS-WBI.

Recently, Takaoka et al. reported a dosimetric comparative study of HS-WBI (intensity-modulated proton therapy [IMPT] vs. helical tomotherapy) [35]. In their study, the hippocampus-Dmean was 11.1 Gy with helical tomotherapy and 7.0 Gy with IMPT (*p* < 0.001). Conversely, the hippocampus-Dmax was slightly higher with IMPT (15.4 Gy) than with helical tomotherapy (14.7 Gy; Table S3). In our study, the hippocampus-Dmean and -Dmax were 7.95 Gy and 11.10 Gy in the BROAD-RT plan. Although the results of our study could not be directly compared to those with the IMPT plan, BROAD-RT seemed to be adequately effective in improving hippocampal dose–volume parameters.

BROAD-RT emerges as a novel and feasible treatment option to facilitate the clinical implementation of HS-WBI. However, BROAD-RT involves low-dose scattering outside the primary treatment field because of the characteristics of the beam pathway with a noncoplanar dynamic swing arc. In our study, the parotid glands-Dmean was slightly higher with BROAD-RT (4.83 Gy) than with Conv-VMAT (4.35 Gy). Because this extremely low-dose scattering had little impact on parotid gland dysfunction [36], the clinical impact of this low-dose scattering is limited. Therefore, we consider that BROAD-RT with OXRAY is an excellent treatment device for planning HS-WBI.

This study has some limitations. First, the small sample size restricts the generalizability of the findings. Second, radiobiological parameters, such as Normal Tissue Complication Probability, could not be used for evaluating the influence of HS-WBI on treatment outcomes because of the lack of well-validated linear quadratic or Lyman–Kutcher–Burman-based Normal Tissue Complication Probability models for the hippocampus. Third, the quality of the treatment plan may have been influenced by the planner’s optimization skills. In particular, in BROAD-RT, which allows for nearly omnidirectional beam selection without the risk of collision with the patient or couch, the optimal beam arrangement may not have been completely considered. However, this also highlights the potential for further improvements in the dose distribution through enhanced planning strategies. Despite these limitations, BROAD-RT using the OXRAY system can reduce the hippocampal dose while maintaining a PTV coverage comparable to that of Conv-VMAT.

## 5. Conclusions

The BROAD-RT for HS-WBI improved the hippocampal dose distribution (Dmax, 11.10 vs. 16.10; Dmean, 7.95 vs. 9.89; Dmin, 7.01 vs. 8.24; V10, 0.42 vs. 39.05; all *p* < 0.01) without worsening the PTV coverage, when compared with Conv-VMAT. In addition, although the beam delivery time of BROAD-RT was longer than that of Conv-VMAT, the total treatment time of HS-WBI with BROAD-RT was sufficiently short for clinical applications.

## Figures and Tables

**Table 1:** Optimization-objective characteristics.

	Mean Volume (Range)
PTV (cm^3^)	1364.69 (1170.65–1439.43)
Hippocampus volume (cm^3^)	6.53 (4.72–8.10)
Eye volume (cm^3^)	16.81 (12.76–21.16)
Lens volume (cm^3^)	0.36 (0.28–0.43)

PTV, planning target volume.

**Table 2:** Paired *t*-test for the comparison between two radiotherapy plans (BROAD-RT vs. Conv-VMAT).

		BROAD-RT	Conv-VMAT	*p*-Values
PTV				
	D98 (%)	27.95 (0.54)	28.21 (0.48)	0.27
	D50 (%)	31.65 (0.26)	31.58 (0.44)	0.62
	D2 (%)	33.55 (0.49)	33.26 (0.66)	0.09
	V30 (%)	95.00 (0.00)	95.00 (0.00)	-
	V35 (%)	0.22 (0.26)	0.17 (0.30)	0.29
	HI	0.18 (0.02)	0.16 (2.23)	0.14
Hippocampus				
	Dmax (Gy)	11.10 (0.61)	16.10 (0.57)	<0.01
	Dmean (Gy)	7.95 (0.20)	9.89 (0.75)	<0.01
	Dmin (Gy)	7.01 (0.19)	8.24 (0.34)	<0.01
	V10 (%)	0.42 (0.34)	39.05 (25.89)	<0.01
Eye				
	Dmax (Gy)	16.17 (1.68)	15.23 (1.58)	0.20
Lens				
	Dmax (Gy)	3.76 (0.16)	4.26 (0.32)	0.13
Parotid glands				
	Dmean (Gy)	4.83 (0.35)	4.35 (0.83)	0.03
Chiasm				
	Dmax (Gy)	34.28 (1.04)	34.66 (1.30)	0.50
Dose count				
	DMU (cGy/MU)	1743.70 (151.44)	1222.71 (280.20)	<0.01
Beam delivery time				
	Time (s)	313.60 (34.91)	202.50 (14.46)	<0.01

BROAD-RT and Conv-VMAT dose–volume parameters are expressed as mean (standard deviation). BROAD-RT, biaxially rotational dynamic radiation therapy; Conv-, conventional; VMAT, volumetric modulated arc therapy; PTV, planning target volume; Vxx, volume receiving xx Gy; Dxx, dose receiving xx% of the volume; DMU, dose monitor unit; HI, homogeneity index. HI was calculated as follows: HI = (D2 − D98)/Dmedian × 100.

## Data Availability

The original contributions presented in this study are included in the article. Further inquiries can be directed to the corresponding author.

## Supplementary Materials

The following supporting information can be downloaded at: https://www.mdpi.com/article/10.3390/cancers17121949/s1, Table S1: Dose constraints for radiotherapy planning; Table S2: Summary of the information on the devices used for treatment planning; Table S3: Summary of the HS-WBI planning study.

## Abbreviations

The following abbreviations are used in this manuscript:

HS-WBI	hippocampal-sparing whole-brain irradiation
WBI	whole brain irradiation
IMRT	intensity-modulated radiation therapy
IMPT	intensity-modulated proton therapy
VMAT	volumetric modulated arc therapy
BROAD-RT	biaxially rotational dynamic radiation therapy
CT	computed tomography
PTV	planning target volume
MU	monitor unit
Conv-	conventional
HI	homogeneity index
Vxx	volume receiving xx Gy
Dxx	dose receiving xx% of the volume
CBCT	cone beam CT
