# Supplementary material for: Dosimetric Study of Biaxially Rotational Dynamic Radiation Therapy for Hippocampal-Sparing Whole Brain Irradiation
Source: Cancers (Basel). 2025 Jun 11;17(12):1949. doi: 10.3390/cancers17121949 (PMC12190859; doi:10.3390/cancers17121949)
Supplement: Supplementary file 1 [file cancers-17-01949-s001.zip › cancers-3664221-supplementary.pdf]

Table S1. Dose constraints for radiotherapy planning

Structure	Index	Objectives
PTV	D98%	>25 Gy
	D95%	>100%
	D2%	<37.5 Gy
Hippocampal	D_{max}	<17 Gy
	D100%	<10 Gy
Chiasm	D_{max}	<37.5 Gy

PTV, planning target volume; Dxx%, dose to xx% of the structure; D_{max}, maximum dose of the structure.

Table S2. Summary of the information on the devices used for treatment planning

	**BROAD-RT**	**Conv-VMAT**
Treatment machine	OXRAY	TrueBeam
Treatment planning system	Raystation	
Calculation methods	Collapsed Cone Version 5.8	
No. of Arc	3	4
MLC width (central) [mm]	2.5	5
MLC width (peripheral) [mm]	5	10
MLC thickness [mm]	110	67
Maximum leaf speed [cm/s]	6.5	2.5
Collimator angle [°]	-	Arc1: 85 (Jaw-Cut X1)<br>Arc2: 95 (Jaw-Cut X2)<br>Arc3: 330<br>Arc4: 30
Couch angle [°]	-	Arc1: 0<br>Arc2: 0<br>Arc3: 310<br>Arc4: 50
Swinging angle [°]	Arc1: 20–50<br>Arc2: 340–310<br>Arc3: 0	-

BROAD-RT, biaxially rotational dynamic radiation therapy; Conv-, conventional; VMAT, volumetric modulated arc therapy; MLC, multileaf collimator.

Table S3. Summary of the HS-WBI planning study

Author/year	Methods of radiotherapy	PTV-D98	PTV-D50	PTV-D2	PTV-V35	PTV-V30	PTV-HI	Hippocampus-Dmax	Hippocampus-Dmin	Hippocampus-Dmean	Hippocampus-V10	Lens-Dmax	Eye-Dmax	(Optic nerve and) Chiasm-Dmax	MU	Treatment time (seconds)
Gondi 2010 [20]	Tomotherapy vs. IMRT	-	-	-	-	-	0.16 vs. 0.30	12.8 vs. 15.3	-	-	-	3.4 vs. 3.8	-	-	-	-
Zieminski 2018 [21]	MCO-VMAT*1 vs. Conv-VMAT	-	-	-	2.7 vs. 2.3	92.7 vs. 87.9	-	15.9 vs. 15.7	7.8 vs. 8.2	9.1 vs. 9.3	21.9% vs. 19.7%	7.0 vs. 7.3	-	-	746.9 vs. 1152.3	-
Wang 2015 [22]	Conv-VMAT vs. IMRT	28.25 vs. 26.02	-	32.33 vs. 32.85	-	-	0.13 vs. 0.22	13.92 vs. 14.11	-	-	-	5.23 vs. 4.66	-	-	-	-
Rong 2015 [23]	Tomotherapy vs. Conv-VMAT	27.5 vs. 26.8	-	32.2 vs. 3.39	-	94.4 vs. 95.5	0.15 vs. 0.22	15.1 vs. 13.6	8.0 vs. 8.6	-	-	3.3 vs. 8.1	8.5 vs. 21.0	33.9 vs. 34.4 (Optic nerve and Chiasm)	-	1080 vs. 150
Yokoyama 2022 [24]	Tomotherapy vs. Conv-VMAT (3arc)	25.34 vs. 28.35	31.60 vs. 32.62	33.07 vs. 34.65	-	-	-	12.63 vs. 14.32	6.15 vs. 6.35	8.02 vs. 7.89	-	3.32 vs. 8.78	12.47 vs. 19.45	-	N/A vs. 2052	1539.9 vs. 166.4
Takaoka 2021 [35]	Tomotherapy vs. IMPT	26.7 vs. 28.1	-	35.2 vs. 33.8	-	-	1.50 vs. 1.28	14.7 vs. 15.4	9.3 vs. 3.8	11.1 vs. 7.0	-	-	32.0 vs. 26.7	31.8 vs. 32.8 (Optic nerve: 30.2 vs. 32.8)	-	-
Present study	BROAD-RT vs. Conv-VMAT (4arc)	27.95 vs. 28.21	31.65 vs. 31.58	33.55 vs. 33.26	0.22 vs. 0.17	95.0 vs. 95.0	0.18 vs. 0.16	11.1 vs. 16.1	7.01 vs. 8.24	7.95 vs. 9.89	0.42 vs. 39.05	3.76 vs. 4.26	16.17 vs. 15.23	34.28 vs. 34.66	1743.7 vs. 1222.7	313.6 vs. 202.5

All values in the table represent mean values.

Abbreviations: BROAD-RT, biaxially rotational dynamic radiation therapy; Conv-, conventional; VMAT, volumetric modulated arc therapy; IMRT, intensity-modulated radiation therapy; IMPT, intensity-modulated proton therapy; PTV, planning target volume; Vxx, volume receiving xx Gy; Dxx, dose receiving xx% of the volume; HI, homogeneity index; MU, monitor unit.

*1: VMAT planned using multi-criteria optimization
